# Assessment of electronic disease early warning system for improved disease surveillance and outbreak response in Yemen

**DOI:** 10.1186/s12889-020-09460-4

**Published:** 2020-09-18

**Authors:** Fekri Dureab, Kamran Ahmed, Claudia Beiersmann, Claire J. Standley, Ali Alwaleedi, Albrecht Jahn

**Affiliations:** 1 Heidelberg Institute of Global Health, Medical School, Ruprecht-Karls-University, Heidelberg, Germany; 2 Institute of Research in International Assistance, Akkon-Hochschule für Humanwissenschaften, Berlin, Germany; 3 World Health Organization, WHO Health Emergencies, Regional Office for Africa (AFRO), Brazzaville, Republic of Congo Republic of the Congo; 4 Center for Global Health Science and Security, Georgetown University, Washington, D.C., USA; 5 Faculty of Medicine and Health Sciences,, University of Aden, Aden, Yemen

**Keywords:** Performance indicators, Assessment, Disease surveillance, Early warning system, eDEWS, Yemen, Outbreak response, Public health emergencies

## Abstract

**Background:**

Diseases Surveillance is a continuous process of data collection, analysis interpretation and dissemination of information for swift public health action. Recent advances in health informatics have led to the implementation of electronic tools to facilitate such critical disease surveillance processes. This study aimed to assess the performance of the national electronic Disease Early Warning System in Yemen (eDEWS) using system attributes: data quality, timeliness, stability, simplicity, predictive value positive, sensitivity, acceptability, flexibility, and representativeness, based on the Centres for Disease Control & Prevention (US CDC) standard indicators.

**Methods:**

We performed a mixed methods study that occurred in two stages: first, the quantitative data was collected from weekly epidemiological bulletins from 2013 to 2017, all alerts of 2016, and annual eDEWS reports, and then the qualitative method using in-depth interviews was carried out in a convergent strategy. The CDC guideline used to describe the following system attributes: data quality (reporting, and completeness), timeliness, stability, simplicity, predictive value positive, sensitivity, acceptability, flexibility and representativeness.

**Results:**

The finding of this assessment showed that eDEWS is a resilient and reliable system, and despite the conflict in Yemen, the system is still functioning and expanding. The response timeliness remains a challenge, since only 21% of all eDEWS alerts were verified within the first 24 h of detection in 2016. However, identified gaps did not affect the system’s ability to identify outbreaks in the current fragile situation. Findings show that eDEWS data is representative, since it covers the entire country. Although, eDEWS covers only 37% of all health facilities, this represents 83% of all functional health facilities in all 23 governorates and all 333 districts.

**Conclusion:**

The quality and timeliness of responses are major challenges to eDEWS’ functionality, the eDEWS remains the only system that provides regular data on communicable diseases in Yemen. In particular, public health response timeliness needs improvement.

## Background

Surveillance is the continuous systematic collection, analysis, and interpretation for actions starting from health planning, implementing interventions and assessing policies and practices in public health [1]. Recurrent epidemics, in some cases resulting in global pandemics, demonstrate the need to strengthen national disease surveillance systems, and prevent onward transmission to other countries. Advances in public health informatics have allowed countries that have experienced recent disasters to develop and implement real time reporting, transmission and processing of epidemiological data for timely detection, verification and prompt public health actions [2, 3].

Yemen has experienced a long period of civil unrest, including several active conflicts, over the past decade [4]. An armed conflict began in March 2015, causing a severe humanitarian crisis for the population [5]. This ongoing conflict has collapsed the essential life services and left 22.2 million people in serious need of humanitarian support [6]. People were forced to settle in temporary settings or host many families in small spaces with high population densities, unsafe water, inadequate food, poor sanitation and lack of basic social and health services that pose significant risk factors associated with potentially life-threatening communicable disease outbreaks leading to increased morbidity and mortality [7, 8]. Rapid detection and prompt response to diseases and epidemics is fundamental during humanitarian disasters particularly in countries with poor disease surveillance mechanisms. Public health surveillance systems become disturbed or overwhelmed to meet needs of a humanitarian emergency, including timeliness and high data quality.

**Fig. 1:**
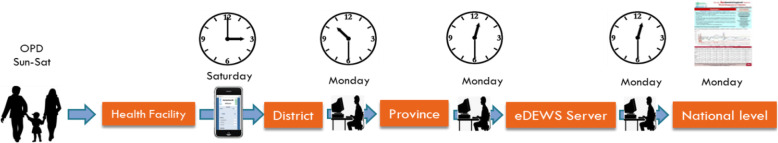
The flow of data in all levels of eDEWS

The Electronic Diseases Early Warning System (eDEWS) is a health facility-based disease surveillance system using an electronic tools and platform for effective data collection, management, analysis and visualizations using dashboard. It was established to strengthen the routine disease surveillance system, mainly in early detection of epidemic-prone diseases, and to thus facilitate rapid responses [3]. eDEWS was initiated in Yemen in March 2013. It started as a pilot project in 4/23 governorates (provinces) with 98 health facilities (sentinel sites). The evaluation of the pilot phase showed that eDEWS can complement the routine disease surveillance system to detect potential outbreaks in a timely way [9]. Currently, eDEWS covers 1982 health facilities in all 333 districts in Yemen. The system began by reporting on 16 communicable diseases, and later increased to include 31 [10]. eDEWS was initially designed as an early warning system, and so can identify the alerts immediately after data entry at peripheral level for timely public health action. At the same time, it sends SMS alerts to all responsible authorities at district, governorate and central (national) levels to be verified and take rapid action. A weekly summary bulletin is also generated. Figure 1 summarizes the flow of data in eDEWS from all levels of health system in Yemen. The health facilities submit weekly summary of prioritized epidemic-prone disease under surveillance to eDEWS system on a weekly basis (every Saturday) using various electronic devices (mobile phones, tablets, laptops etc.). The various health system units ensure validation of submitted information in eDEWS platform within 24 h of a health facility-reporting deadline. The validated data is immediately accessible to the national level eDEWS team for final review, translated into information by eDEWS built-in automated analytics modules, and published on a national level dashboard immediately available to relevant stakeholders on Monday afternoon. It eases the quick transformation of data into actionable information and knowledge-translation for better planning and evidence-based decision making [9]. Thus, the early warning system is needed to cover the gap during crises; currently, it has been integrated into the routine disease surveillance system that was disrupted during the conflict [3, 7, 11]. Data on various aspects of the eDEWS and its relation to routine surveillance systems in Yemen are not sufficiently addressed in literatures. This study aimed to assess the performance of the eDEWS system and explore to what extent this program is useful and able to identify early alerts of epidemics in Yemen during the conflict. Therefore, the findings are intended for policy makers to improve the performance of the health system in Yemen and it will be useful for other similar setting.

## Methods

**Fig. 2:**
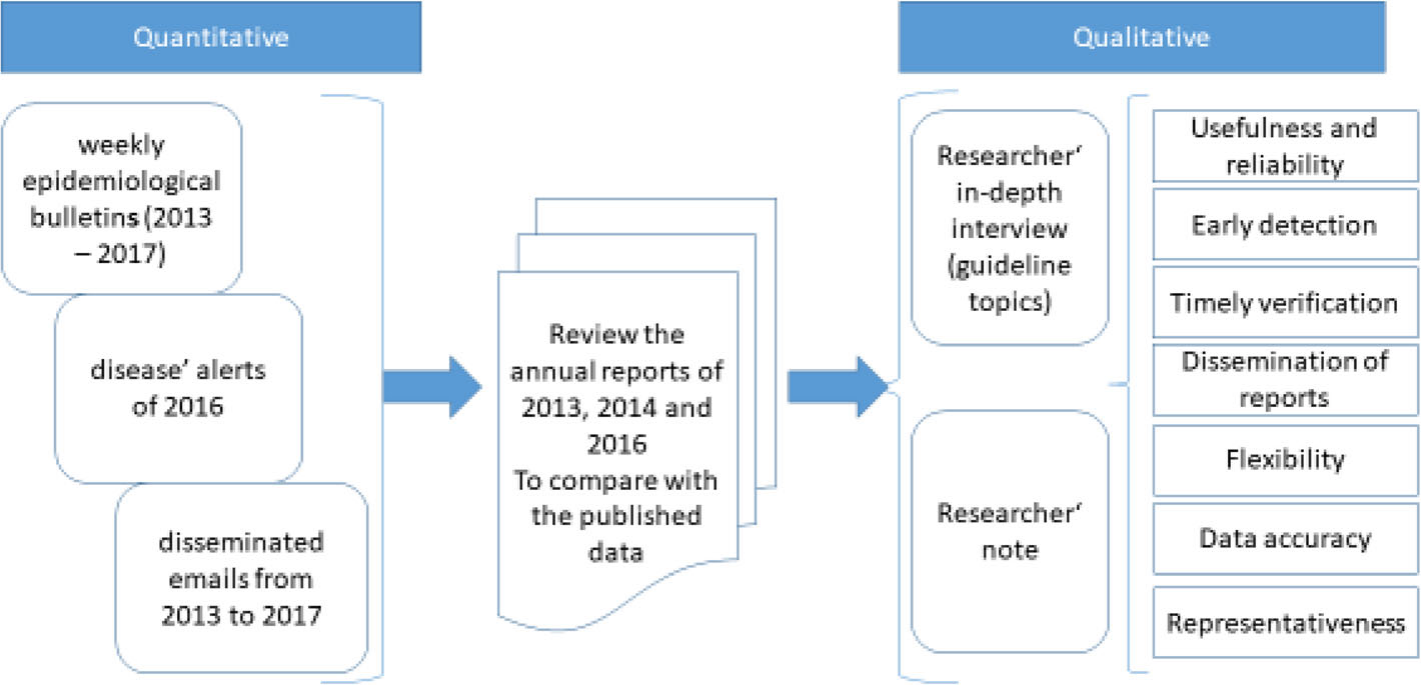
Flowchart of mixed method research approach

We performed a mixed methods study that occurred in two stages: first, the quantitative part then followed by qualitative part using in-depth interviews. The qualitative method was carried out in a convergent strategy. Both quantitative and qualitative datasets are given equal weight in terms of analysis, Fig. 2 shows the flow diagram of the used methods. The Centres for Disease Control & Prevention (CDC) standard indicators were used in this assessment [12], to describe the following system attributes: data quality (reporting, and completeness), timeliness, stability, simplicity, predictive value positive, sensitivity, acceptability, flexibility and representativeness see Table 1.

**Table 1:** The CDC standard performance indicators to assess the usefulness of surveillance system [12]

Indicator	Definition
**Data quality**	depends on the completeness and validity of eDEWS data, and the accuracy of its reports.
**Timeliness**	refers to the speed or interval between steps in the eDEWS. The time interval between any two sequential steps can be assessed.
**Simplicity**	refers to the simple structure and ease in applying the procedure to improve the timeliness of the eDEWS.
**Positive predictive value (PPV)**	reflects the proportion of confirmed cases or alerts from the condition under surveillance. eDEWS allows for the calculation of a PPV at the level of case detection depending on the number of alerts generated and the proportion of confirmed alerts as truly under surveillance.
**Sensitivity**	The sensitivity of a surveillance system can be considered on two levels. At the level of case reporting, sensitivity refers to the proportion of cases of a disease detected by the surveillance system. Sensitivity also can refer to the system’s overall ability to detect outbreaks, including the ability to monitor changes in the number of cases in a population over time.
**Acceptability**	indicates the willingness of health workers and partners to participate in the surveillance system.
**Flexibility**	means the ease with which a) information or conditions can be changed as needed, b) eDEWS can accommodate a new disease, c) changes can be made in case definitions, and d) variations can be made in reporting sources.
**Representativeness**	defines disease occurrence over time and the characteristics of a covered population.

### Verification process in eDEWS

In the eDEWS, an alert was defined as an early signal about a targeted epidemic-prone disease, condition or event of public health importance, which can alert the early stages of an outbreak. An alert notification is generated by eDEWS when the number of reported cases reach or exceed the defined alert threshold for epidemic prone diseases under surveillance.

eDEWS system detects alerts based on an application of a defined thresholds for each disease under surveillance to the weekly data it receives from health facilities. Furthermore, the immediate notification alerts are encouraged even outside weekly reporting, and notified to the eDEWS system through immediate reports by health system staff and other information sources. Other information sources may include volunteers participating in community based surveillance (CBS), who have already been oriented on the agreed indicators (lay case definitions), or any other representatives from community, who have been trained to detect unusual public health events, and report them to the next level in the health system.

All generated alerts need to be triaged, investigated further and verified as to whether they represent a real event (true alert) or are false alarms that do not precede significant excess cases (false alerts). Only true alerts are then investigated through formal investigations that may include review of clinical cases, line listing of cases, sampling for laboratory confirmation, identification of potential sources of transmission, contact tracing, active case search and limited containment interventions.

Alerts of prioritized epidemic-prone diseases are notified to the surveillance teams through the eDEWS system, which then triggers the alert verification process. Once the alert is assessed for risks and verified as a true alarm, field investigations are conducted, including sampling and laboratory investigations. As a result, if an alert meets case definition, reach or exceed alert thresholds, and confirmed by lab investigations, they are classified as “true positive”. Some alerts are verified to be true alarm upon initial verifications such as met case definition and alert threshold but later determined to be false upon further laboratory investigations, were considered as “false positive”. Conversely, alerts that do not meet case definition or reach alert threshold, and lab samples are tested negative, are discarded as false alarm during the verification process by response teams and are classified as “false alerts”.

### Study procedure and data collection

#### The quantitative data

The quantitative part was predominantly extracted from the weekly epidemiological bulletins (2013–2017), all disease’ alerts of 2016, disseminated emails from 2013 to 2017, and annual eDEWS reports. Three spreadsheets were developed to collect and enter the data based on the required variables; the first sheet developed to collect data from the weekly epidemiological bulletins (2013–2017), reflecting the reporting rate of health facilities, the frequency of diseases, distribution of cases according age and sex, and the total alerts generated per week for each disease of the list. All reported diseases in this system were clinically diagnosed using the case definition, and were classified into five groups. The first group was respiratory diseases or airborne diseases; the second group included digestive system diseases or water/food borne diseases; the third group was vector borne diseases; the fourth group included vaccine-preventable diseases; and the last group included all other infectious diseases such as chicken pox, brucellosis, schistosomiasis, rabies, HIV/AIDS, tuberculosis (TB), scabies and Guinea worm. All alerts that generated in 2016 were extracted from the eDEWs dashboard in the second spreadsheet, it was including the disease’ alerts, name of health facility and governorate, number of cases, time of reporting, verification and investigation, as well as the mean of verification. The third spreadsheet was developed to collect data on the time and date of disseminated bulletin from emails that sent to health partners from 2013 to 2017to compare the actual date of sending the bulletin against the required date of sending.

Finally, three existing annual reports of 2013, 2014 and 2016 were reviewed to assess the quality of data in comparison with the published data in the eDEWS bulletins.

#### The qualitative data

This component of the study focused on individual interviews with 11 key informants responded out of 20 key informants. They were invited from those routinely involved in the core and support functions of the eDEWS and who have acquired adequate knowledge and experience on functioning at the central and peripheral levels of the health system. Five eDEWS staff and surveillance officers at governorate level, and six key informants from central level from Ministry of Public Health and Population (MoPHP) and NGOs (Table 2). The informants were purposefully selected based on their experience and involvement in either disease surveillance or use the eDEWS in their routine work. Two guidelines were prepared (semi-structured questionnaires) which focused on the performance indicators of eDEWS. The first guideline prepared to target those who are working directly in eDEWS and the second one focused on people who use the eDEWS information in their humanitarian actions [13]. The guidelines were modified based on the pilot testing and comments of the ethical committee. The interviews were either conducted by the first author using Skype, or via another trained interviewer by the author in Yemen, in order to reach people without internet access. Interviews were digitally recorded and transcribed verbatim in English by the first author. Accuracy of each transcribed interview was verified with the digital recording by the authors and revisions made as necessary.

**Table 2:** Respondents at the various health system levels

Position	Frequency	Percentage
National Level	6	54.6%
MoPHP	3	27.3
Int. NGOs	3	27.3
Governorate Level:	5	45.4%
Health Managers	3	27.3
Health facility staff	2	18.1
Total	11	100

### Data entry and analysis

The quantitative data was entered, cleaned and analysed using the Statistical Package for Social Sciences (SPSS version 25). Frequencies, percentages, mean and timeliness were produced from the analysis.

The transcripts of in-depth interviews were uploaded into NVivo 12® to assist with data organization, coding and constructing queries. Codes were developed by the main interviewer based on performance indicators of the CDC and the interview guide [12]. A deductive method was used to assign the data to categories data based on the theme for analysis. An inductive approach was used as well to identify emerging themes across the categories. The codes were revised by an expert on qualitative data analysis and discussed with a senior supervisor from our team.

## Results

### Data quality

#### Reporting

**Fig. 3:**
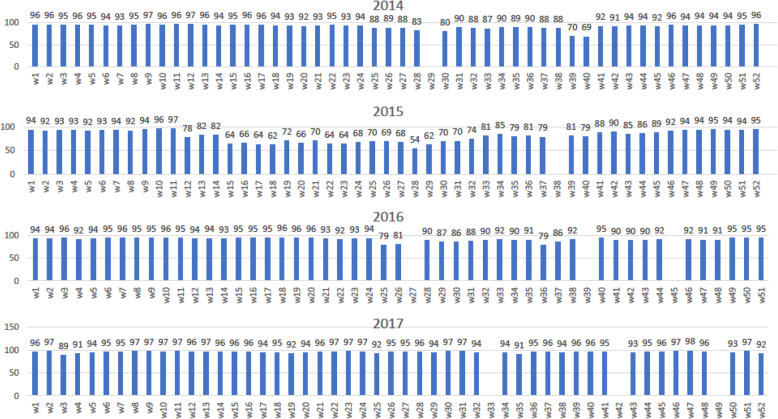
The total reporting rate of sentinel sites by week from 2014 to 2017

Figure 3 shows the reporting rate in eDEWS by week from 2014 to 2017. The average reporting rate was more than 90% in all years except in 2015, when it was around 80%.

#### Accuracy

Quality of data is a crucial aspect of any health information system. Four of the interview respondents believed that the manual data management needs improvement since discrepancies have been found between tables and figures in the same bulletin. Other problems in data accuracy included false positive diagnosis. Informants highlighted factors such as poor understanding of case definitions, poor recording of cases, poor monitoring and evaluation mechanism due to weak health system and security situation as potentially contributing to the issues observed with data accuracy.

#### Completeness

According to the informants, weekly data submitted by health facilities using a mobile application would have to be complete by default since the eDEWS system does not accept incomplete forms. Completeness of eDEWS data was obviously noted by key informant from another vertical surveillance system such as polio or measles program in the country. However, there were some discrepancies between eDEWS data and disease-specific programs which reflect differences in the way cases are counted, rather than issues of completeness. Key informant mentioned that eDEWS provides information about disease in total numbers and not by cases; in this way, the same patient may visit multiple health facilities and thus be counted multiple times in eDEWS.

*“Compare to other alternatives, eDEWS data are of good quality. It is not perfect but good enough and can be improved [ … … ]”* **Informant # 1**

*“It is difficult to say eDEWS is precise because of the current conflict situation and the weak health system in Yemen, it gives approximate data about the situation, and it is good to reflect the situation”* **Informant # 4**

### Timeliness

In principle, the time taken to transmit surveillance data from health facility to national level is two working days, including the time for data validation by both district and governorate levels, in order to ensure high quality of data for data analysis and report generation at national level.

Table 3 shows the time interval between date of reporting in eDEWS and the first rapid action taken by eDEWS staff to verify the alerts (first response of diseases type A that need an immediate action within 24 h). Data shows 21% of alerts were verified within 24 h from the reporting time, and 42% were verified between 24 and 48 h of the reporting date and 21% were verified after 48 h. Only 16% of alerts time remained unknown.

**Table 3:** Time interval between reporting and investigation day in 2016

Time interval	Number of alerts	Percentage
Response within 24 h	791	21%
Response within 48 h	1553	42%
Response more than 48 h	777	21%
No date found on responses	599	16%
Total alerts in 2016	3721	100%

The majority of informants indicated that there was a delay in timely reporting in eDEWS, although all informants recognized the eDEWS function of early detection of diseases. Eight participants observed that the verification process is very slow compared to the required action for immediate alerts.

Key informants reported that it is challenging to verify all eDEWS generated alerts within 24–48 h and not later than 72 h, which is another eDEWS surveillance indicator. Factors contributing to the difficulty of verifying alerts included the ongoing crisis and associated financial situation that has disrupted health infrastructure, worsened ground security and resulted in many health staff remaining unpaid.

*“From my experience, usually the response team does not respond in the first 24 hours, maybe it is not everywhere but due to the difficulties and the available infrastructure, it may take more than 24 hours to respond”.* **Informant # 5**

Table 4 shows that the mean delay time of the weekly bulletin dissemination has increased in the last two years. It was 4 days in 2013, 2.8 days in 2014 and 0.15 days in 2015; however, in 2016 and 2017 the mean delay time increased to 9 days.

**Table 4:** Mean time delay in data dissemination in eDEWS

Year	Number of published Bulletin	Delay in days		Mean	Std. Deviation
		Minimum	Maximum		
2013	32	3	9	4.06	1.242
2014	50	0	10	2.80	1.654
2015	46	0	5	0.15	0.788
2016	49	5	30	9.55	4.912
2017	48	3	22	9.00	4.048

Respondents reported that expansion of eDEWS program from 200 health facilities to 2000 health facilities has significantly increased burden of reporting on health staff resulting in delays in alert verification and processing of information for data analysis, reporting and dissemination of information to stake holders.

*“Honestly speaking, there are many issues that challenge the eDEWS from publishing the Bulletin [in a] timely [manner]. But, I will not talk about them since there are very sensitive and political issues”* **Informant # 2**

### Simplicity and stability

eDEWS was reported to be stable, and able to collect and manage data without any major disturbances. It is available 24/7, and focal points have access to the system any time when needed.

“*the percentage of time the system is operating fully about 99%, only once or twice per year the system goes down and each time it does not take more than an hour to fix it*”. **Informant # 11**

In case of internet outage, the focal person at the district or governorate level receives data by phone call and enters it using his computer. All focal persons at health facilities received training on data collection and data entry using the electronic form, which can be accessed by mobile phone or via computer.

*“eDEWS is simple and has a basic form to enter the data, it is not complicated to use the form, if you have something happened it give you an immediate alert and staff has to confirm it in the system directly, so it is easy to use. The only problem is the internet connectivity and now we have a solution to make it fillable form offline, fill the form and save it then when internet is available can be sent automatically.”* **Informant # 1**

### Positive predictive value (PPV)

Table 5 shows that eDEWS central database generated 2075 SMS alerts in 2013, of which 1561 SMS alerts were verified as true alerts (positive predictive value [PPV] of 75%). Of these true alerts, six were confirmed as outbreaks. In 2016, eDEWS had the lowest PPV of 72% (28,476 true alerts were verified out of 39,624 generated alerts), while in 2015 and 2017 the system had the highest PPV of 95% and five outbreaks were detected in each year.

**Table 5:** Total positive predictive value (PPV) by year

No	Indicators	2013	2014	2015	2016	2017
1	No. alerts	2075	4281	5321	39,624	126,555
2	No. true alerts	1561	3583	5046	28,476	120,637
3	positive predictive value (PPV)	75%	84%	95%	72%	95%

### Sensitivity

All listed events were reported on a weekly basis, using standardized national case definitions to diagnose the cases. Disease trends can be monitored and the change in numbers of cases is easily observed. The system can generate SMS alerts if the number of cases exceeds pre-determined thresholds. However, calculation of sensitivity was difficult using the available data. It is difficult in emergency situation to have complete data and it is difficult to do laboratory or field investigation for all alerts.

eDEWS plays a role in early detection of cholera and diphtheria epidemics. All respondents agreed that eDEWS able to detect outbreaks early because of its innovative and simple method of using mobile phones in reporting.

*“eDEWS can detect outbreak but can be better, for example cholera was detected by eDEWS*”. **Informant # 3**

*“I think the alarm of the eDEWS was the guide not only for detect cases but even for the death cases and everything becomes clear now”* **Informant # 5**

*“The weekly diseases surveillance reveals the spread of any new case for any outbreak earlier and report about it.”* **Informant # 9**

### Acceptability

eDEWS is widely accepted by health personnel working in support of the system. The willingness of eDEWS staff to continue working for the system, and maintain a high rate of reporting, despite all the challenges in the field provides further evidence of its acceptability. Other surveillance systems and partners from different organizations showed willingness to support eDEWS.

*“Our work in eDEWS is very exhausting but we are going forward not backward despite the challenges and obstacles our work is continuous every day and night no interruption in work”.* **Informant # 6**

*“You know eDEWS is a Ministry of Public Health structure system and partners every time express their willing to supporting and help MoPHP in the surveillance system for eDEWS”* **Informant # 2**

### Flexibility

Most of the participants agreed that eDEWS is a flexible surveillance system. It is a national program owned by MoPHP and WHO, easy to maintain, and flexible enough for expansion to new health facilities or customizations, for example if new priority diseases need to be added. However, a few participants think that eDEWS may take a long time for program scale-up compared to other non-electronic vertical programs, due to procurement requirements of electronic devices and the need for training personnel on electronic devices for data collection and transmission.

*“WHO and MOPHP owned the code and maintain the system and improved it anytime. It is flexible enough for new changes and modification.”* **Informant # 1**

*“It takes very long time to be able for any change or expansion because when you add health facilities you need to provide a mobile phone and train them on software and reporting to do this is not easy.”* **Informant # 7**

### Representativeness

The eDEWS is present in all the 23 governorates of Yemen and covers all the 333 districts. The total number of health facilities involved in eDEWS was 1982 by end of 2017. This represents 37% of the total number of health facilities in the country (5316). 88% of health facilities in Sanaa (the capital city of Yemen) are included in eDEWS, followed by Aden governorate which has a 79% coverage rate. Amran, Albaidha and Taiz have the lowest coverage rates of 21, 22 and 26% respectively in 2017. Males and females from all age categories were present in the eDEWS data. Around 35% of patients were between age 15–44 years old in each year from 2013 to 2017, and 53% were female patients.

*“we are working in all governorates of Yemen we cover around 1,982 health facilities in all districts, at least there is one health facility in a district. The selection of health facilities was done by the governorate health offices they selected the main health facilities in the districts to reflect the morbidity situation in the catchment and be representative as possible”.* **Informant # 8**

## Discussion

This study addresses the function of Yemen’s electronic diseases early warning system (eDEWS), as a national disease surveillance system, from 2013 to 2017, during an on-going conflict. Disease surveillance is an important component of public health for tracking potential epidemics, monitoring interventions, and informing health policy [14]. This study is one of the few performed in Yemen and the Eastern Mediterranean Region to assess an eDEWS, based on CDC standard indicators and identify the system’s usefulness during Yemen’s ongoing complex emergency.

Key findings showed that eDEWS is a resilient and useful system, and despite the conflict, the system is still functioning. Data quality and response timeliness were somewhat problematic, since only 21% of all eDEWS alerts were verified in the first 24 h of detection in 2016. However, these gaps did not affect the system’s ability to identify outbreaks in the current fragile situation. This study’s findings show that eDEWS data is representative.

Data completeness remains a significant challenge for many national surveillance programs [15], however, the high level of completeness in eDEWS is ensured due to a mobile software electronic data collection process, and the system’s validation function that ensures submission of complete reports by health facilities. Global evidence shows that the use of electronic reporting systems contributes to good data quality in terms of availability, timeliness, reliability, and completeness [16]. The high reporting rate in eDEWS reflects the data completeness and system acceptability for all health staff and partners involved in the system, this findings are in agreement with the findings of similar evaluations conducted in Sana’a Governorate [17].

Despite the high rate of report completeness in eDEWS, manual data management and analysis poses significant risk to data accuracy in generated epidemiological reports, which may affect data usage in decision making [18]. Manually managing and analysing data is time intensive, increases workload, and poses significant risk of human error in data compilation and analysis. The shortage in laboratory confirmation and dependence on case definitions for diagnosis is one of the challenges affecting the quality of data, particularly in an emergency as seen in other countries with similar circumstances [19], which may compromise data quality and the accuracy of disease surveillance data used by decision-makers. We recommend switching from manual to automated data analysis processes within the existing online database system. This approach can drastically decrease dependency on manual methods and help to avoid errors and delays [20].

Immediate public health action is always required in public health surveillance following the effective reporting of health facility information. Mayad et al. (2019) argue for the perfect timeliness of eDEWS at 100% [17]. However, our study shows that only 21% of alert responses for diseases requiring immediate action occurred within the requisite 24-h window (out of a total of 3721 such alerts), thus highlighting a gap in the response timeliness. A response delay during outbreaks increases the burden of morbidity and mortality [20]. For example, in 2016, only 31% of the cholera cases received a response within the first 24 h of the eDEWS alert notification [21]. The delays in verification of data has a substantial effect on the detection process. As another example, during the cholera outbreak in 2016, there were many alerts of acute watery diarrhoea in Al Baidha governorate several weeks before declaring the outbreak in Sana’a, however, these alerts were not verified in a timely manner, and thus early warning of the possible spread of the disease was not delivered [21]. Response timeliness remains a problem even in many higher-income countries, e.g., in the USA, a study found a significant difference in response delay times compared to the standard immediate response time for Category II vaccine-preventable diseases in West Virginia [22].

In Yemen, where public health efforts are often implemented by non-governmental partners, delays in dissemination of weekly information may be one reason for delayed partner intervention (especially in water, sanitation and hygiene [WASH] interventions), thus reducing surveillance usefulness due to a missing link between data collection and public health action [23]. This study revealed that dissemination delays increased over time from 2.8 days in 2014 to 9.0 days in 2016 and 2017, the eDEWS was expanded quickly from 100 health facilities in 2013 to 1982 in 2017, thus data processing was affected by the overwhelming amount of data received each week. All key informants interviewed in this study confirmed the delay in the dissemination of the weekly eDEWS bulletin. In Syria, the average delay for publishing information was 24 days for the Early Warning and Response System (EWARS) based in Damascus, while in Turkey, the average delay was 11 days for Early Warning and Response Networks (EWARNs) [24].

Identifying the barriers and challenges facing a surveillance system is a critical step for improving performance; our study revealed timeliness as a particularly chronic issue with eDEWS in Yemen [25]. Rapid staff turnover, the security situation, limited resources for alert response, health staff motivation, refresher training needs, limited technical capacities, logistics, issues of internet connectivity and a lack of financial resources all likely contribute to poor timeliness. Despite all these challenges, eDEWS nevertheless demonstrated its appropriateness even during the conflict and its on-going utility for detecting new emerging outbreaks. Cordes KM et. al recommends at-risk countries to invest in such systems, as early warning, alert, and response networks (referred to by WHO as EWARN) have been useful sources of information where no other data were available during many emergencies [26].

A Positive Predictive Value (PPV) reflects the ability of the system to detect true outbreak. Having low false positive alerts, especially in 2015 and 2017 in Yemen, reflected the program’s effectiveness in detecting outbreaks. Therefore, detected outbreaks were generally true with an average PPV of more than 95% (range 95–100%). This in line with a systematic review study comparing an electronic surveillance system with a paper surveillance method that showed that electronic surveillance has moderate to excellent utility compared with conventional surveillance methods [27]. A low PPV for a surveillance system leads to wasted resources and time due to an unnecessary investigation of every reported case [28]. The eDEWS is the only system capturing data on epidemic prone diseases during the humanitarian emergency of Yemen with lack of laboratories, therefore, sensitivity is difficult to assess [29].

Data obtained from the system shows that eDEWS is able to detect changes over time since data can be supported by field investigation and laboratory testing. eDEWS is very useful in monitoring disease trends in Yemen’s current situation. This study shows that Yemen’s eDEWS is a reliable surveillance system with the possibility of contributing to the timely detection and monitoring of diseases. For example, in 2016, eDEWS monitored the trends of dengue fever cases in the system on a weekly basis, and there were a total of five confirmed dengue outbreaks (Aden, Lahj, Mareb, Hajjah and Al-Hodeida). The eDEWS was useful in locating outbreaks in unusual geographic locations, for example, cholera and dengue fever were reported for first time in Sanaa in 2016 [13]. Even when a public health surveillance system has low sensitivity, it can still be useful in trend monitoring as long as the sensitivity remains reasonably constant and change is notable [28].

Population representation in any surveillance system is influenced by access to the health facilities as well as sex and age groups [30]. In Yemen, eDEWS data are regularly used to provide national estimates of the incidence and prevalence of infectious diseases and guidance for required interventions. The eDEWS is used by only 37% of all health facilities in the country, however, this represents 83% of all functional health facilities [31]. All age groups are represented in eDEWS data; and one-third of the patients were between 15 and 44 years. Approximately 53% of the registered patients in the health facilities were women, a similar finding was reported in a study on the representativeness of an online nationwide surveillance system for influenza in France [32].

Measuring eDEWS’s usefulness and acceptability is the main attribute of an evaluation to demonstrate functionality and ensure the system’s sustainability [33]. Acceptability is a cross-cutting measure of surveillance usefulness. It can be measured by several indicators such as the percentage of reporting, completeness and responses by surveillance staff and relevant stakeholders. Results show that various partners are supporting eDEWS in the field, and many donors trust the system to identify new emerging outbreak in the country. The evidence showed that increasing the health staff and field health partners’ transparency and knowledge of the system’s processes will increase the surveillance system’s accessibility [34]. Many key informants did not agree that eDEWS is a flexible system since they believed that eDEWS needs more time to achieve change. However, flexibility is not a matter of time, but rather the ability to adapt to changes in risks and information input [33].

The major limitation of the study revolves around the difficulty to get full data for the period from 2013 to 2017, we used eDEWS data predominantly extracted from the epidemiological bulletins. Moreover, we found only three annual reports of eDEWS, and the annual reports of 2015 and 2017 were not produced by the program. It was difficult to extract full data on alerts from the program dashboard for all targeted years, and the only available full data was for 2016. For consistency, all incomplete data on alerts was excluded. Due to the lack of data on routine surveillance, it was impossible to compare eDEWS data with other Yemeni routine surveillance data sources for better assessment of the quality of eDEWS information.

In emergency situation as in Yemen, it is difficult to have complete data and it is difficult to do laboratory or field investigation for all alerts. Therefore, we tried to use the available data in the system to identify the PPV for the system as whole. The sensitivity of the eDEWS system could not be determined exactly on the basis of available data. Therefore, this paper focused on the application of case definitions to determine whether there have been any factual changes.

## Conclusion

In conclusion, although the quality and timeliness of responses are major challenges to eDEWS’ functionality, the eDEWS remains the only system that provides regular data on communicable diseases in Yemen. Improving response timeliness may be matters requiring the attention of the local and international partners. Beyond that, the pioneering experience from Yemen, including the relative resilience and robustness of eDEWS, may also inform health agencies and authorities in similarly fragile, conflict-prone and deprived setting, on how to cope with the threat of infectious diseases and epidemics through outbreak detection and an enhanced rapid response during a conflict.

## Data Availability

The data was obtained from the published weekly bulletin; the reports were sent by Ministry of Health via email. The alerts data of 2016 were obtained from the MoPHP directly. Formal permission was granted by the authority of the MoPHP in Yemen to use the eDEWS data.

## Abbreviations

eDEWS: Electronic Disease Early Warning System
MOPHP: Ministry of Public Health and Population
NGO: Non-Governmental Organization
PPV: Positive Predictive Value
WHO: World Health Organization
